# Partial substitution of the ureter using a double short segments of the ileum following the monti procedure

**DOI:** 10.11604/pamj.2015.20.270.5954

**Published:** 2015-03-19

**Authors:** Mounir Lahyani, Nabil Jakhlal, Fouad Bakloul, Tarik Karmouni, Khalid Elkhader, Abdellatif Koutani, Ahmed Ibn Attya Andaloussi, Ismail Bezza, Mohamed Elouazni, Lhssan Ifrine, Abdelkader Belkouchi

**Affiliations:** 1 Department of Urology B, Ibn Sina Hospital, Rabat, Morocco; 2 Department of Surgery A, Ibn Sina Hospital, Rabat, Morocco

**Keywords:** Ureter, stenosis, treatment, ileum, substitution, monti procedure

## Abstract

The partial substitution of the ureter using a pediculated double short segments of the ileum is a technique used to re-establish ureteral transit and preserve the renal unit, following the resection of extensive ureteral lesions. Standard surgical procedure for an ileoureteroplasty consists of isolating an ileal duct of equal or greater length than the ureteral defect and interposing it in the urinary tract in an isoperistaltic direction. Monti described a surgical technique that allows for the creation of catheterizable stomas in continent urinary diversions, using the Mitrofanoff principle. These passageways were created from one or several 2.5 cm long ileal sections by means of their detubulization and transverse retubulization.

## Introduction

Long ureteric defects may occur as a result of iatrogenic ureteral injuries, recurrent pelvi-ureteric junction obstruction, chronic inflamatory diseases such as tuberculosis, retroperitoneal fibrosis and ureteral carcinoma. Several techniques were described to compensate this defect by intrinsic urinary tissue such as direct anastomosis, psoas hitch, Boari flap, transuretero-ureterostomy and renal autotransplantation. In some occasions the ureteral defect is too long to the degree that extra urinary tissue is needed to overcome it. Accordingly, other alternatives have been tried such as artifi cial ureteral substitutes e.g. Gore-Tex tube graft [1] which showed disappointing results in contrast to the promising ones that were achieved with the use of pedicled bowel grafts [2]. The most commonly used technique is that of ileal ureter replacement which was first described by Schoemaker in 1906 [3] and gained wide acceptance later on. One of the modifications of this technique was the application of Yang-Monti principle [4, 5] which allowed the creation of a long tube from short bowel segment after its reconfiguration. It was applied for ureteral replacement first in dogs [6] then clinically [7] in few reports.

## Patient and observation

**Figure 1:**
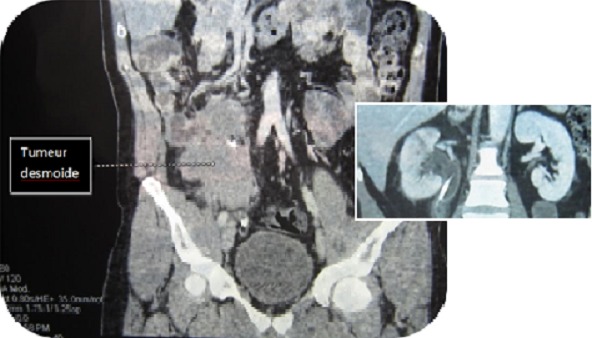
Scan showing abdominal mass with ureterohydronephrosis

A.S 45 year old man with a desmoid tumor right story, recently diagnosed by computed tomographic examination (Figure 1) for the occurrence of multiple right renal colic. Physical examination was unremarkable. Renal function was normal and urine culture was negative. Initially, a double j stent was performed to compensate for the right ureterohydronéphrosis. Three months later, surgical removal by median laparotmy was performed. A long portion of the iliolumbar ureter was sclerotic, so we decided to make a ureteral replacement.

### Technique

**Figure 2:**
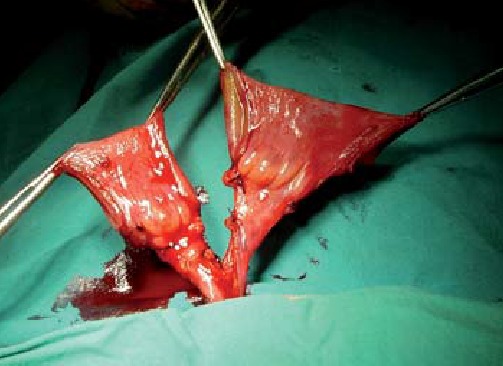
Isolation of 2 ileal segments on their mesenteric pedicle

**Figure 3:**
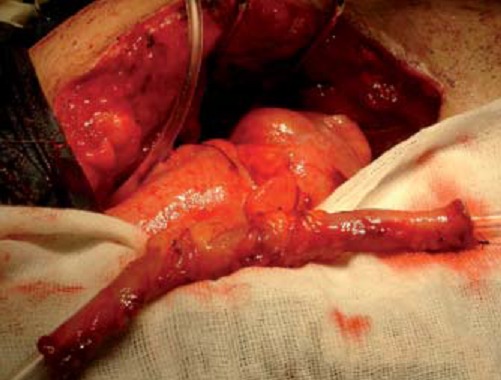
Tubularization of the incised ileal segments

Isolation of 5 - 7 cm of the terminal ileum on their vascular bed was performed according to the length required to compensate the ureteral defect. Then, according to the Yang-Monti principle, the isolated ileal segment was further subdivided into 2 equal parts (each 2.5 cm in length) with preservation of the individual blood supply (Figure 2). The continuity of the ileum was reestablished. Each ileal segment was incised at its longitudinal axis close to the mesenteric border. Unfolding of the incised segments with suturing of their adjacent ends were performed and resulted in the formation of an intestinal plate 2.5 cm wide and 10-12 cm. This plate was tubularized around 16F Nelaton catheter using 4/0 absorbable sutures (Figure 3). Then we realized a spatulisation both proximal and distal ends of the ileum, before establishing a uretero-ileal anastomosis without tension and using two hemi-running 3/0 absorbable sutures. A double j protection stent will be kept for 4 weeks.

## Discussion

Nowadays, iatrogenic ureteral trauma showed an increased incidence as one of the causes of ureteric stricture [8, 9]. This could occur as a result of direct injury or focal ischemia to the ureter [10]. Ileal ureter replacement became now an established procedure for ureteric reconstruction especially after the urologists gained more experience in bowel surgery [11]. Several studies reported encouraging results regarding surgical outcome and renal function [2, 9], however common drawbacks were reported mostly attributed to the absorbing and secreting criteria of the involved ileal segment such as hyperchloremic metabolic acidosis and excess mucus production and also to the wide caliber refluxing ileal ureter with subsequent progressive dilatation, functional obstruction and recurrent urinary tract infections [9, 12]. So, to overcome these complications and to improve the surgical outcome, the Yang-Monti principle was applied as a modification of original simple ileal ureter replacement, which allowed the reconstruction of long tube from a reconfigured short bowel segment.

Intraoperatively, we used 2 ileal segments. Each ileal segment provided a tube of about 6 cm length after reconfiguration. We found that this technique is safe as it was not associated with mortality or significant morbidity. During follow-up, no complaint of excess mucus production was observed which was also noticed by other authors that used the same procedure [7]. This observation is considered an advantage of this modification and most probably due to the marked reduction in the size of the secreting surface area in comparison to simple ileal ureter that may be associated with mucous obstruction in some cases [12]. Moreover, hyperchloremic metabolic acidosis did not occur in our case, however it was reported by various studies using simple ileal ureter in varying percentages [8, 9]. Absence of metabolic disorders among our case can be explained by proper selection of patient (serum creatinine = 12 mg/l) and reduction of the size of absorbing surface area.

**Figure 4:**
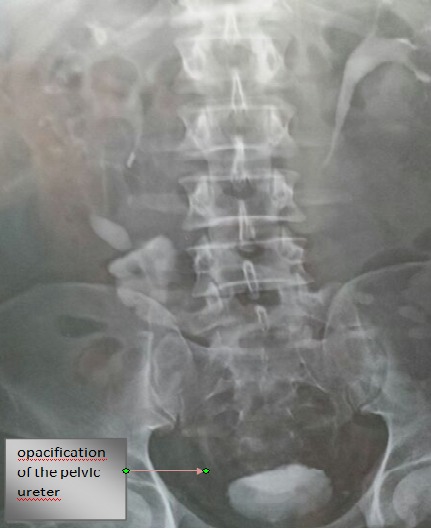
Urography postoperative control

UIV showed improvement of split renal function without urinary obstruction (Figure 4), so we found that ileoureteral anastomosis can be done successfully with no worry about organic or functional obstruction in carefully selected cases. This was facilitated by the diminished caliber of the ileal tube after its reconfiguration which added another advantage to Yang-Monti modification.

## Conclusion

We advice the use of Yang-Monti principle in ileal ureter replacement as it consumes a double short bowel segment with less mucus production and absence of metabolic abnormalities. It also leads to creation of a long tube of reasonable diameter. Moreover, it is a safe technique without significant morbidity that permits a durable preservation of renal function.
